# Supplementary material for: Self Fourier shell correlation: properties and application to cryo-ET
Source: Commun Biol. 2024 Jan 16;7:101. doi: 10.1038/s42003-023-05724-y (PMC10791666; doi:10.1038/s42003-023-05724-y)
Supplement: Supplementary file 2 — Supplemental Material [file 42003_2023_5724_MOESM2_ESM.pdf]

Supplemental Information

Supplementary Note 1 Applicability of the SFSC to CTF modified signals

Microscopy images are typically corrupted by imaging artifacts. In cryo-EM, this is modeled by the contrast transfer function (CTF) which is the Fourier transform of the point spread function of the microscope. We therefore discuss the effects of these modifications on the estimation of the FSC by SFSC. Often, the forward model may be written as:

$$\hat{y} = \hat{c} \cdot \hat{x} + \hat{\epsilon} \tag{17}$$

where $\hat{x}$ is the Fourier transform of the underlying signal, $\hat{c}$ is the CTF effect, and $\hat{y}$ is the Fourier transform of the corrupted observation. Given two images of the same signal affected by the same CTF, the FSC can be readily used to estimate the SSNR, but it should be noted that it will be an estimate of the SSNR for the corrupted signal $\tilde{x} = \mathcal{F}^{-1}\{\hat{c} \cdot \hat{x}\}$, and not the clean signal x. Similarly, the SFSC can also be used to estimate the SSNR of the corrupted signal assuming the measurement follows the properties assumption 1 and assumption 2. We illustrate this with two simulated examples. The first example is for a typical CTF in cryo-EM images, described by:

$$\text{CTF}(\xi) = \sqrt{1-w^2}\sin(-\pi\lambda z\|\xi\|^2 + C_s\lambda^3\|\xi\|^4\pi/2 - \alpha) - w\cos(-\pi\lambda z\|\xi\|^2 + C_s\lambda^3\|\xi\|^4\pi/2 - \alpha), \tag{18}$$

with $w = 0.1$, $\lambda = 2.51$ pm, $z = 2.8$ µm, $C_s = 2.0$ µm, and $\alpha = 0.87$. The second example is for a CTF that resembles a tilt series image in cryo-ET (see Supplementary Note 7), described by:

$$\text{CTF}(\xi) = \sin(\alpha + \|\xi\|^2/z), \tag{19}$$

with $\alpha = 3/4$ and $z = 5$. We generate noisy, CTF corrupted images following eq. (17). The FSC, SFSC and spherically averaged power spectrum are then computed for each image Figure S1. As expected, the oscillations of the CTF modulate the correlation profile in both the FSC and SFSC. Importantly, the SFSC still approximates the FSC well. In these scenarios, the traditional resolution value obtained from a threshold may not be meaningful as we expect the FSC to oscillate. Most importantly, while the CTF may change the reported resolution value, the Wiener filter computed using the SFSC of a CTF modified signal is still a statistically optimal filter for denoising.

We note that if the CTF is not radially symmetric, then the CTF corrupted signal should not be expected to satisfy radially symmetric assumptions. In this case, both the FSC and SFSC are poor estimators of the SSNR. However, when applied to reconstructions from a collection of CTF corrupted images with a random and uniform distribution of poses, the reconstructions will have approximately radially symmetric noise and variance even if the images do not.

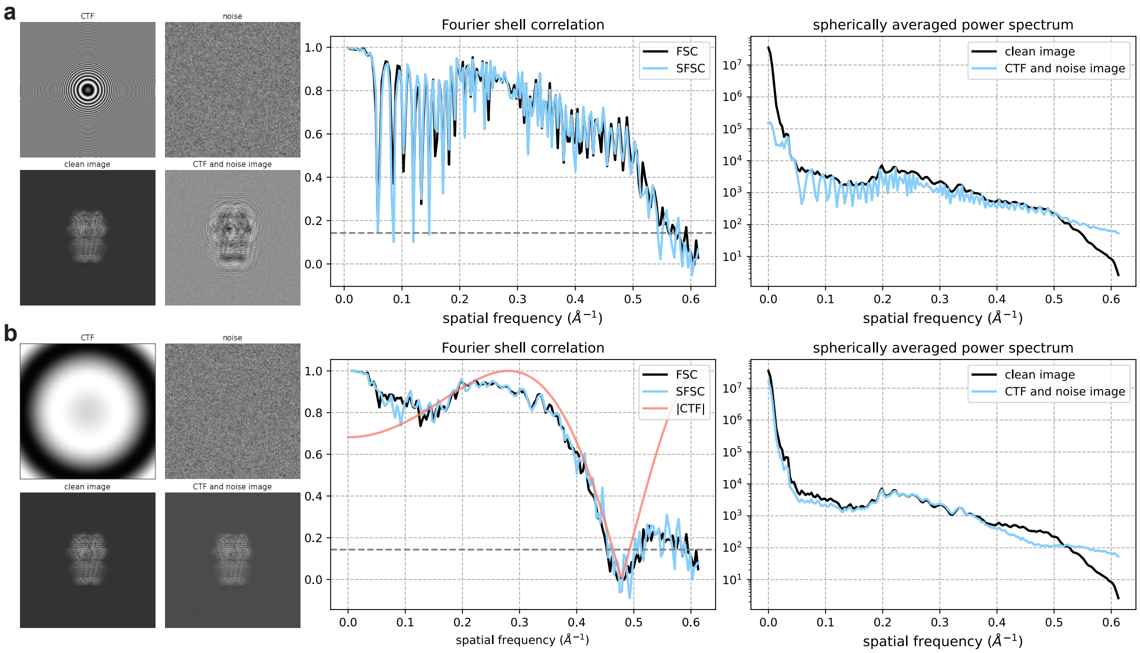

Figure S1: Effect of the CTF on the FSC and SFSC. Clean image is a projection of EMD-11657 ($N \times N = 360 \times 360$, pixel size = 0.812Å). Additive Gaussian noise was generated with SNR = 15 and $B_{noise} = 10\text{Å}^2$. **(a)** Results for image formed using CTF in eq. (18). **(b)** Results for image formed using CTF in eq. (19). Also plotted is the absolute value of the radial CTF.

Supplementary Note 2 Comparison of downsampling methods

The foundation of the SFSC is that a real space measurement can be downsampled by decimation to generate multiple approximations of the measurement whose correlation can then be computed in Fourier space. In our proposed downsampling scheme (see Figure 1), the number of measurements to be compared is equal to the spatial dimensions of the signal (*e.g.*, 3 pairs for a 3-D signal). In the procedure proposed by Koho et al. [8], there are $\binom{2^{\text{dim}}}{2}$ pairs to be compared. There are two main disadvantages of splitting in a checkerboard-like pattern compared to only splitting along one dimension at a time, as proposed in this work. First, the variance of the noise in the downsampled measurements is scaled by 2^{dim}, where dim is the number of dimensions split across. This relation is derived in the following section. Second, the Nyquist frequency will be reduced to half of the original. We demonstrate both of these effects using the 2-D case of an image in Figure S2.

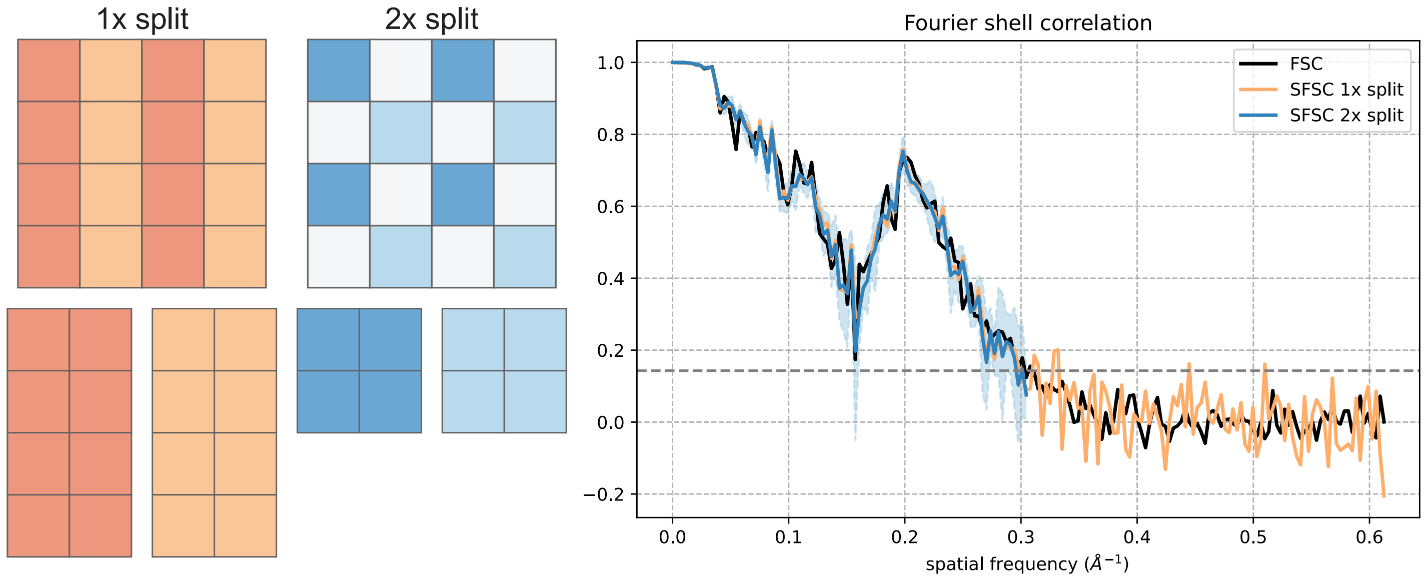

Figure S2: SFSC computed from an image downsampled by splitting along 1 and 2 dimensions. The pixel diagram depicts one pair out of two for the 1× split and one pair out of six for the 2× split. The input image used for the FSC and SFSC is from Figure 3B. The SFSC from the 1× split image is reported as the average of both pairs. The SFSC from the 2× split image is reported as the average of the six combinations and is plotted with an error envelope representing the standard deviation of the SFSC from all combinations.

Supplementary Note 3 General relation between FSC and SFSC

To relate the SFSC to the FSC, we need to relate the DFT of the decimated signals to the DFT of the original signal. We first show this relation for a signal split along one dimension, as advocated in this work, and then provide an example for a signal split along two dimensions, as done for images in the original splitting scheme by Koho et al. [8], to demonstrate how it extends to a signal split along multiple dimensions.

Let x be a 1-D signal of length N. The DFT of x is defined as $\hat{x}[k] = \sum_{n=0}^{N-1} x[n]\omega_N^{nk}$, where $\omega_N = \exp(-2\pi i/N)$. The signal x can be split into even index terms $x_e[n] = x[2n]$ and odd index terms $x_o[n] = x[2n+1]$ for $n \in \{0, \ldots, (N/2)-1\}$. We want to relate $\hat{x}_e[k]$ and $\hat{x}_o[k]$, the DFTs of x_e and x_o, to the DFT of the original signal $\hat{x}[k]$. The relation can be seen by splitting the DFT of x into the sum of the even and odd terms:

$$\begin{aligned}\hat{x}[k] &= \sum_{n=0}^{\frac{N}{2}-1} x[2n]\omega_N^{2nk} + \sum_{n=0}^{\frac{N}{2}-1} x[2n+1]\omega_N^{(2n+1)k} \\ &= \sum_{n=0}^{\frac{N}{2}-1} x[2n](\omega_N^{nk})^2 + \omega_N^k \sum_{n=0}^{\frac{N}{2}-1} x[2n+1](\omega_N^{nk})^2.\end{aligned} \tag{20}$$

Next, we use that $\omega_N^2 = \exp(-2\pi i/(N/2)) = \omega_{N/2}$ to get:

$$\hat{x}[k] = \sum_{n=0}^{\frac{N}{2}-1} x[2n]\omega_{N/2}^{nk} + \omega_N^k \sum_{n=0}^{\frac{N}{2}-1} x[2n+1]\omega_{N/2}^{nk}. \tag{21}$$

Applying the definition of the DFT to the right side of the equation yields:

$$\hat{x}[k] = \hat{x}_e[k] + \omega_N^k \hat{x}_o[k]. \tag{22}$$

Importantly, $k = 0, \ldots, N-1$, and $\hat{x}_e$ and $\hat{x}_o$ are $N/2$ periodic[1]. We can then independently relate $\hat{x}_e[k]$ and $\hat{x}_o[k]$ to $\hat{x}[k]$ as follows:

$$\hat{x}_e[k] = (\hat{x}[k] + \hat{x}[k+N/2])/2 \tag{23}$$

$$\hat{x}_o[k] = (\hat{x}[k] - \hat{x}[k+N/2])/\left(2\omega_N^k\right). \tag{24}$$

Equation (23) and eq. (24) form the framework of our analysis presented in Section 2.2, and describe the relation between the DFTs of a 2-D or 3-D measurement that has been split into alternating voxels along one dimension.

The analysis above holds for a signal split along multiple dimensions as well, since the DFT can be applied along subsequent dimensions. Specifically, we are referring here to the checkerboard-like splitting pattern from [8]. Suppose now that x is a 2-D signal (*i.e.*, an image). If the signal x is split into even and odd index terms along both dimensions, then we have that:

$$\hat{x}_{e,e}[k_1, k_2] = (\hat{x}[k_1, k_2] + \hat{x}[k_1 + N/2, k_2] + \hat{x}[k_1, k_2 + N/2] + \hat{x}[k_1 + N/2, k_2 + N/2])/4 \tag{25}$$

$$\hat{x}_{o,o}[k_1, k_2] = (\hat{x}[k_1, k_2] - \hat{x}[k_1 + N/2, k_2] - \hat{x}[k_1, k_2 + N/2] + \hat{x}[k_1 + N/2, k_2 + N/2])/\left(4\omega_N^{(k_1+k_2)}\right), \tag{26}$$

where e denotes even and o denotes odd indexing for each dimension of x, and k_1 and k_2 are the frequency indexes of $\hat{x}$. In the case of a noisy measurement, we are interested in the relation between the SFSC from the downsampled measurements using this splitting scheme and the SSNR. Following the arguments presented in Section 2.2, if both assumptions on the signal and noise are met, then:

$$\begin{aligned}\mathrm{ESFSC}_{\text{2-D}}(r) &:= \frac{\mathbb{E}\left[\left\langle \hat{y}_{e,e}, \hat{y}_{o,o} e^{-2\pi i\langle a, k/N\rangle}\right\rangle_r\right]}{\sqrt{\mathbb{E}[\|\hat{y}_{e,e}\|_r^2]\,\mathbb{E}[\|\hat{y}_{o,o} e^{-2\pi i\langle a,k/N\rangle}\|_r^2]}} \\ &= \frac{\lambda^2(r)}{\lambda^2(r) + 4\sigma^2(r)}.\end{aligned} \tag{27}$$

For the general case of decimating into even and odd terms over multiple dimensions, we see that:

$$\mathrm{ESFSC}(r) = \frac{\lambda^2(r)}{\lambda^2(r) + 2^{\dim}\sigma^2(r)}. \tag{28}$$

[1]This well known recursive identity is at the core of the fast Fourier transform algorithm.

Thus, when splitting along multiple dimensions, there is a scaling of 2^{dim} on the noise variance compared to a scaling of 2 from splitting once as proposed in this work. From eq. (28), the relation between the EFSC and ESFSC split over multiple dimensions is:

$$\mathrm{EFSC}(r) = \frac{2^{\mathrm{dim}}\,\mathrm{ESFSC}(r)}{1 + (2^{\mathrm{dim}} - 1)\,\mathrm{ESFSC}(r)}. \tag{29}$$

We show in Figure 3A that the SFSC will yield an underestimate of the FSC if the correction in eq. (29) is not applied. For the 2-D case in Figure 3A, the SFSC was computed using our splitting scheme. Thus eq. (29) is equal to eq. (12).

Supplementary Note 4 FSC with a phase shifted input

We consider the effect of a translation between real space signals when computing the FSC. A translation is naturally induced between pairs of decimated measurements when computing the SFSC and must be corrected. For example, as noted in [8], when an image is decimated in a checkerboard-like pattern, each downsampled image pair is offset by a single pixel in each dimension. Here we show that if the power spectrum of the signal is approximately spherically symmetric, a translation between the inputs to the SFSC, and more generally the FSC, leads to a signal-independent quantity unique for the 1-D, 2-D and 3-D case.

Continuing with the 2-D case, the decimated images represent the same area as the original image, but with half the length for each split dimension. This leads to an effective pixel size of twice the original. With respect to the original image, we expect the translation between the downsampled image pairs to be:

$$y_2(p) \approx y_1(p-a), \tag{30}$$

where $a = [1/2, 0]^T$ for an image split along one dimension, or $a = [1/2, 1/2]^T$ for an image split along two. A translation of 1/2 reflects the increase in pixel size. If the power spectrum of the image decays fast, then the adjacent pixels are indeed correlated. Here we consider the signal to be continuous, with its Fourier transform defined as $\hat{y}(\xi) = \int y(p)\exp(-2\pi i\,\langle \xi, p\rangle)dp$, where ξ is the frequency variable. A translation in real space is equivalent to a phase shift in Fourier space. It follows that:

$$\hat{y}_2(\xi) = \mathcal{F}\{y_2(p)\} \approx \mathcal{F}\{y_1(p-a)\} = \hat{y}_1(\xi)\exp(2\pi i\langle a, \xi\rangle). \tag{31}$$

That is, we expect the spectrum of image y_2 to be approximately equal to the spectrum of image y_1 multiplied by a phase shift. The continuous analogue of the FSC can be expressed as:

$$\mathrm{FSC}(r) = \frac{\int \mathrm{Re}\left(\overline{\hat{y}_1(\xi)}\hat{y}_2(\xi)\right) d\xi}{\sqrt{\int |\hat{y}_1(\xi)|^2\ d\xi \int |\hat{y}_2(\xi)|^2\ d\xi}}. \tag{32}$$

From eq. (30), $\hat{y}_1$ and $\hat{y}_2$ are approximately equal after a phase shift. We can then rewrite the FSC as:

$$\mathrm{FSC}(r) = \frac{\int \mathrm{Re}\left(\overline{\hat{y}_1(\xi)}\hat{y}_1(\xi)e^{2\pi i\langle a,\xi\rangle}\right) d\xi}{\sqrt{\int |\hat{y}_1(\xi)|^2 d\xi \int |\hat{y}_1(\xi)e^{2\pi i\langle a,\xi\rangle}|^2 d\xi}}. \tag{33}$$

If the power spectrum of y is approximately spherically symmetric, then we then have that:

$$\mathrm{FSC}(r) = \frac{|\hat{y}_1(\xi)|^2 \int e^{2\pi i\langle a,\xi\rangle} d\xi}{|\hat{y}_1(\xi)|^2 \sqrt{\int d\xi \int |e^{2\pi i\langle a,\xi\rangle}|^2\ d\xi}} = \frac{\int e^{2\pi i\langle a,\xi\rangle} d\xi}{\sqrt{2\pi 2\pi}} = \frac{1}{2\pi}\int e^{2\pi i\langle a,\xi\rangle} d\xi. \tag{34}$$

For the 2-D case, the inner product can be written as: $\langle a, \xi\rangle = \|a\|\|\xi\|\cos(\phi) = \|a\| r\cos(\phi)$. Since the integral is over the ring, we can reparametrize ϕ, the angle between a and ξ, so that $\cos(\phi) = \sin(\theta)$. We then get that:

$$\mathrm{FSC}_{\text{2-D}}(r) = \frac{1}{2\pi}\int_0^{2\pi} e^{2\pi i\|a\|\, r\sin(\theta)}\ d\theta. \tag{35}$$

In this form, the FSC is equivalent to a scaled zeroth order Bessel function of the first kind, $J_0(q)$, defined as:

$$J_0(q) = \frac{1}{2\pi}\int_0^{2\pi} e^{iq\sin(\theta)}\ d\theta. \tag{36}$$

Comparing eq. (35) to eq. (36), we see that $\mathrm{FSC}_{\text{2-D}}(r) = J_0(2\pi\|a\|r)$. We show in Figure 3A that failing to account for the induced phase shift in a 2-D image reduces the SFSC to eq. (36) which no longer matches the FSC. For the 3-D case, under the similar assumption of a spherically symmetric power spectrum, evaluating the integral in eq. (34) yields:

$$\mathrm{FSC}_{\text{3-D}}(r) = \frac{\sin(2\pi\|a\|r)}{2\pi\|a\|r}, \tag{37}$$

which is equivalent to a scaled and normalized sinc function. Finally, in the 1-D case, without accounting for the translation, we simply have that the FSC reduces to $\exp(-2\pi ik/N)$. Apart from describing a necessary correction needed for computing the SFSC, these results emphasize that the inputs to the FSC must be carefully aligned. Otherwise, the output from the FSC might not reflect the signal to noise ratio, but rather the deterministic and signal-independent functions described above.

Supplementary Note 5 Estimation of the SSNR without the FSC

In Section 2.2 we describe the assumptions under which the SFSC provides an estimate of the FSC. For some scenarios, if both Assumption 1 and Assumption 2 hold, we can estimate σ^2, the variance of the noise, directly from the high frequencies of the power spectrum. In particular, this is true for high frequency shells when the signal spectrum has decayed and the noise level dominates the power spectrum (*i.e.*, the SSNR is small). If the spherically averaged power spectrum does not appear flat at high frequencies, the noise variance cannot be estimated from the power spectrum and a different approach is needed. Estimating the noise is a standard part of the cryo-EM pipeline and is essential for the whitening transform described in Section 2.3. In this work we show the noise variance can be estimated from regions of noise or the difference of half maps from 3-D reconstructions. If one has access to the noise variance, the following simpler and more direct approach can be used to obtain the SSNR:

$$\mathrm{SSNR}(r) \approx \frac{\mathrm{PS}(y)(r) - \sigma^2(r)}{\sigma^2(r)} \approx \frac{\lambda^2(r) + \sigma^2(r) - \sigma^2(r)}{\sigma^2(r)} = \frac{\lambda^2(r)}{\sigma^2(r)}, \tag{38}$$

from which the EFSC can also be computed as:

$$\mathrm{EFSC}(r) = \frac{\mathrm{SSNR}(r)}{1 + \mathrm{SSNR}(r)}. \tag{39}$$

We show in Figure S3 that the SFSC from a half map and the FSC determined using a noise region of the half map for eq. (38) are approximately equal. We additionally show that if two half maps have been computed, the SSNR, and therefore the FSC, can be estimated just as well from the difference of half maps. Thus, this property is not intrinsic to just the SFSC. Nonetheless, the FSC still has the advantage of being scale invariant and can be applied even when there is ambiguity in the scale between the two measurements.

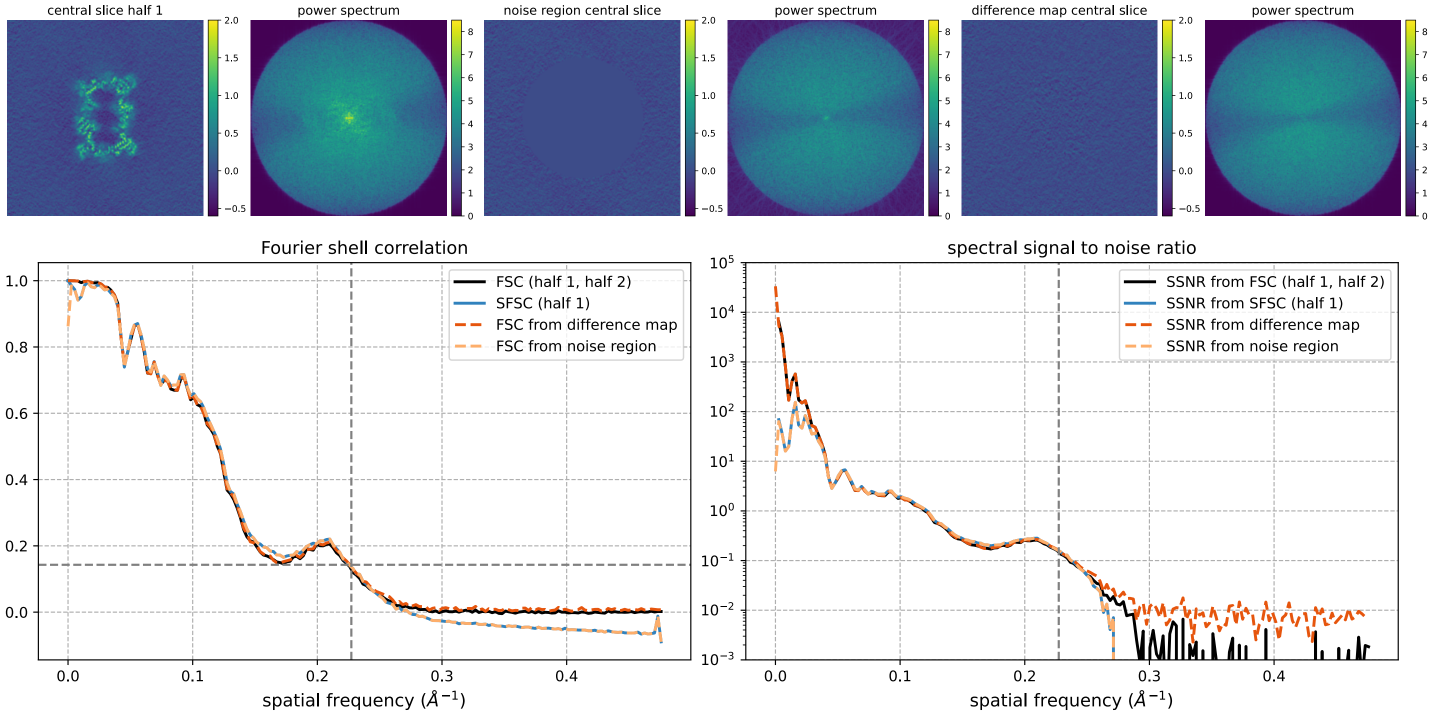

Figure S3: Comparison of the FSC from half maps and the SSNR estimated from measurement noise. The FSC and SSNR are computed from EMD-24822 by four different methods and show similar profiles.

Supplementary Note 6 Remarks on frequency filtering for the SFSC

To avoid aliasing when downsampling a signal, the standard approach is to first low-pass filter the original signal [42]. However, frequency filtering should not be applied when computing the SFSC as both the low and high frequency terms of the original signal are needed to correctly estimate the FSC. This can be directly seen in the DFTs of the downsampled signals which are modified by $\pm(\hat{x}[k+N/2]+\hat{\epsilon}[k+N/2])$, the higher frequency terms. If an ideal low-pass filter was applied to the original signal such that all frequencies $k > N/4$ are set to zero, the resulting SFSC would equal 1 at all frequencies, regardless of the SSNR. Similarly, if the original signal was high-pass filtered such that all frequencies $k < N/4$ are set to zero, the SFSC would equal -1 at all frequencies. Thus, in order to accurately estimate the FSC from the SFSC, frequency filtering of the original signal should be avoided. The upsampling procedure in Section 2.4 used to compute the SFSC for measurements without decaying power spectrum effectively creates a low-pass filter. However, since the measurement must first be whitened for the upsampling procedure to work, we can compensate for the necessary high frequency noise terms since the variance of the noise has been set to 1.

Noise estimation from reconstructed volumes

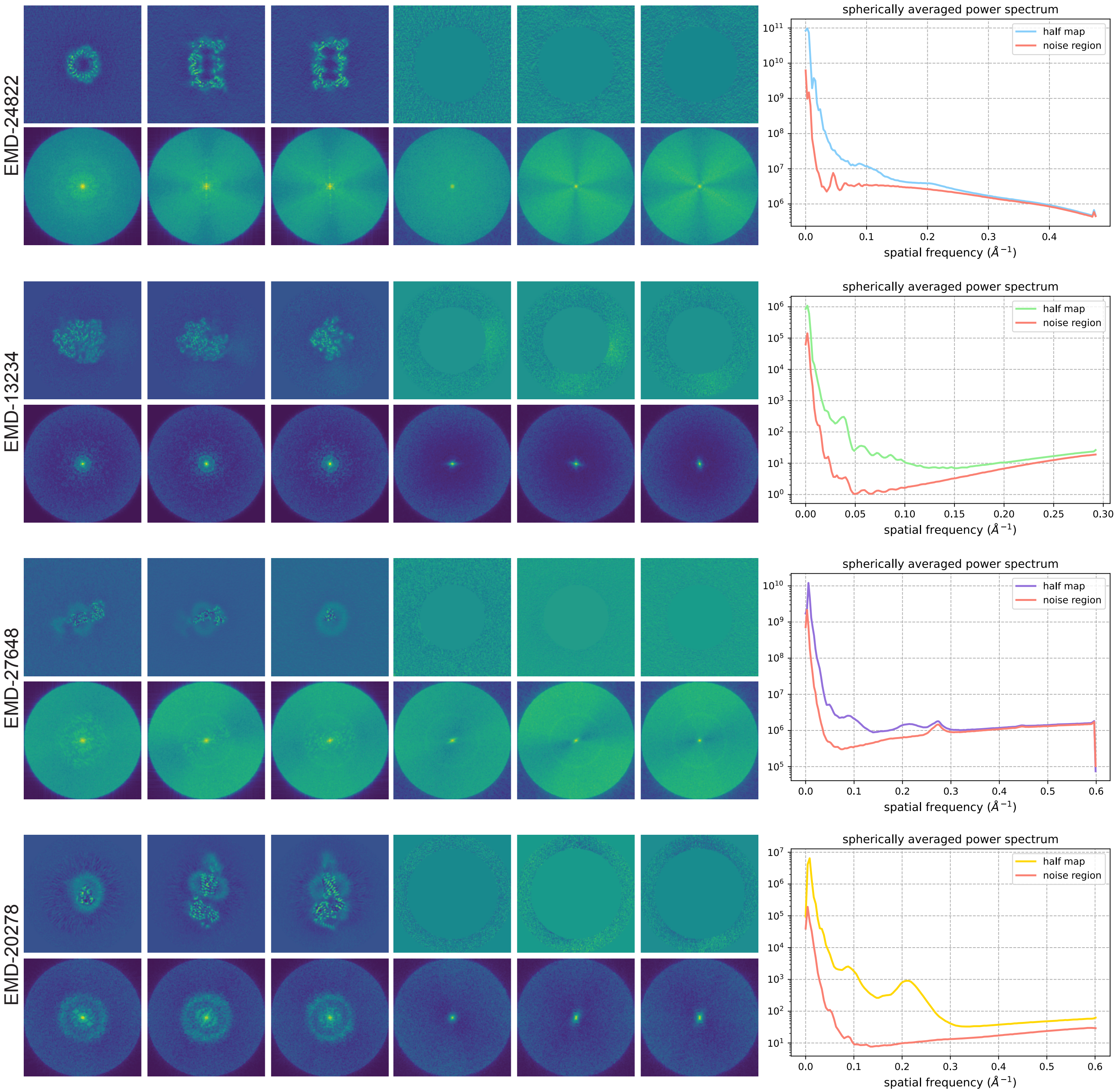

Figure S4: Central slices of the raw half maps, the regions containing only noise, and their corresponding power spectrum (below each image) for the four structures in Figure 4.

Supplementary Note 7 CTF and denoising for tomograms

In Figure S5, we visualize the CTF from a tilt series image and the tomographic reconstruction of EMD-15056 [31]. The power spectrum of the full size tilt series image clearly displays Thon rings. However, the downsampled image used for reconstruction only shows one zero crossing of the CTF. The Wiener filter denoised tomogram and a comparison to the cryo-CARE denoised tomogram is shown in Figure S6.

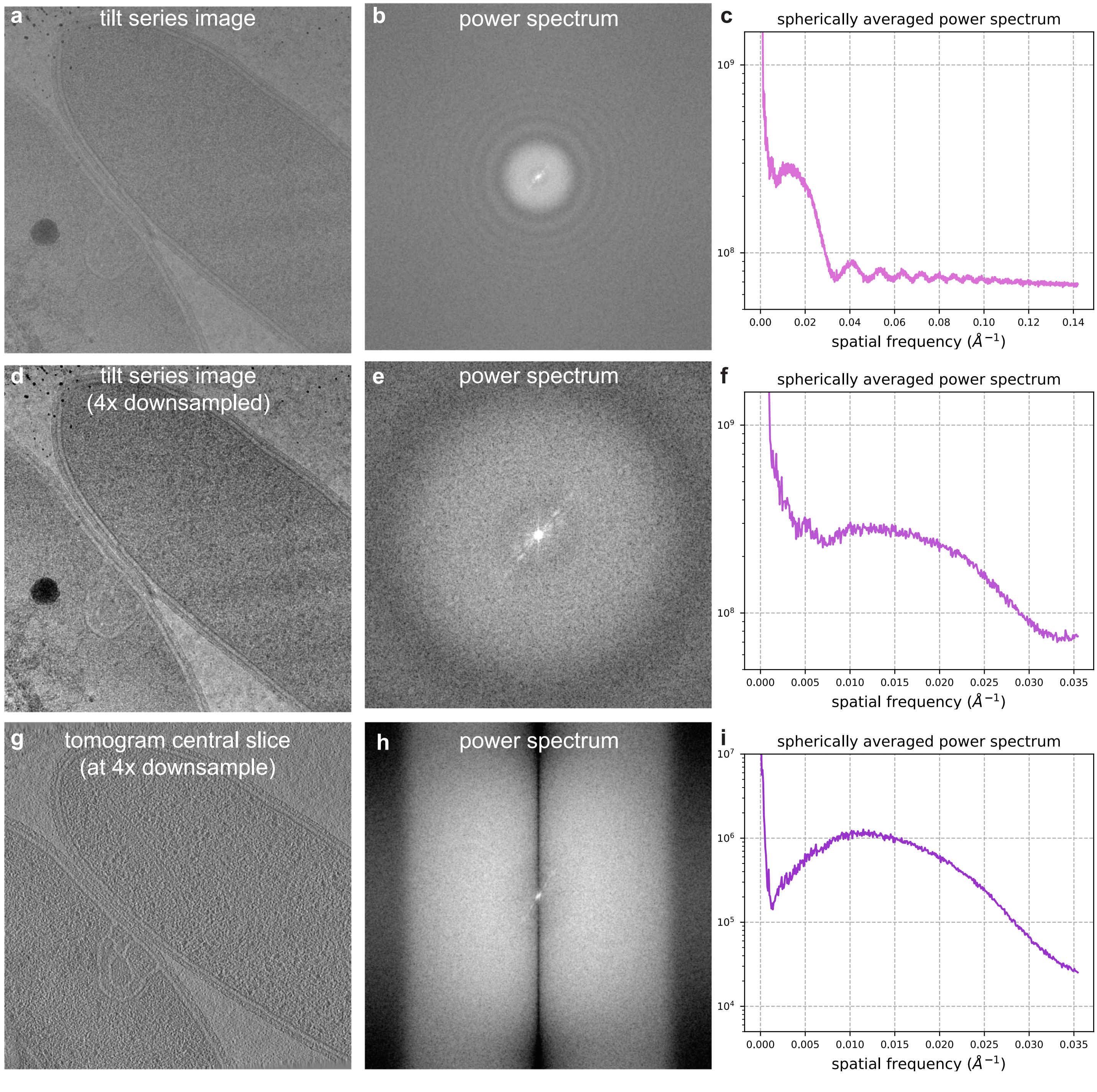

Figure S5: CTF visualization for tilt series and reconstruction. **(a)** Motion corrected tilt series image from EMPIAR-11058 ($N \times N = 3712 \times 3712$, pixel size $= 3.52$Å). **(b)** Power spectrum of tilt series image. **(c)** Spherically averaged power spectrum of tilt series image. **(d)** Tilt series image in **(a)** downsampled 4× as done in the reconstruction pipeline. **(e)** Power spectrum of the downsampled tilt series image. **(f)** Spherically averaged power spectrum of the downsampled tilt series image. **(g)** Central slice of the reconstructed tomogram. **(h)** Power spectrum of a central slice of the reconstructed tomogram. **(i)** Spherically averaged power spectrum of the reconstructed tomogram central slice. The tilt series image and downsampled version used for reconstruction clearly show the CTF. However the CTF effects are less visible in the reconstructed tomogram.

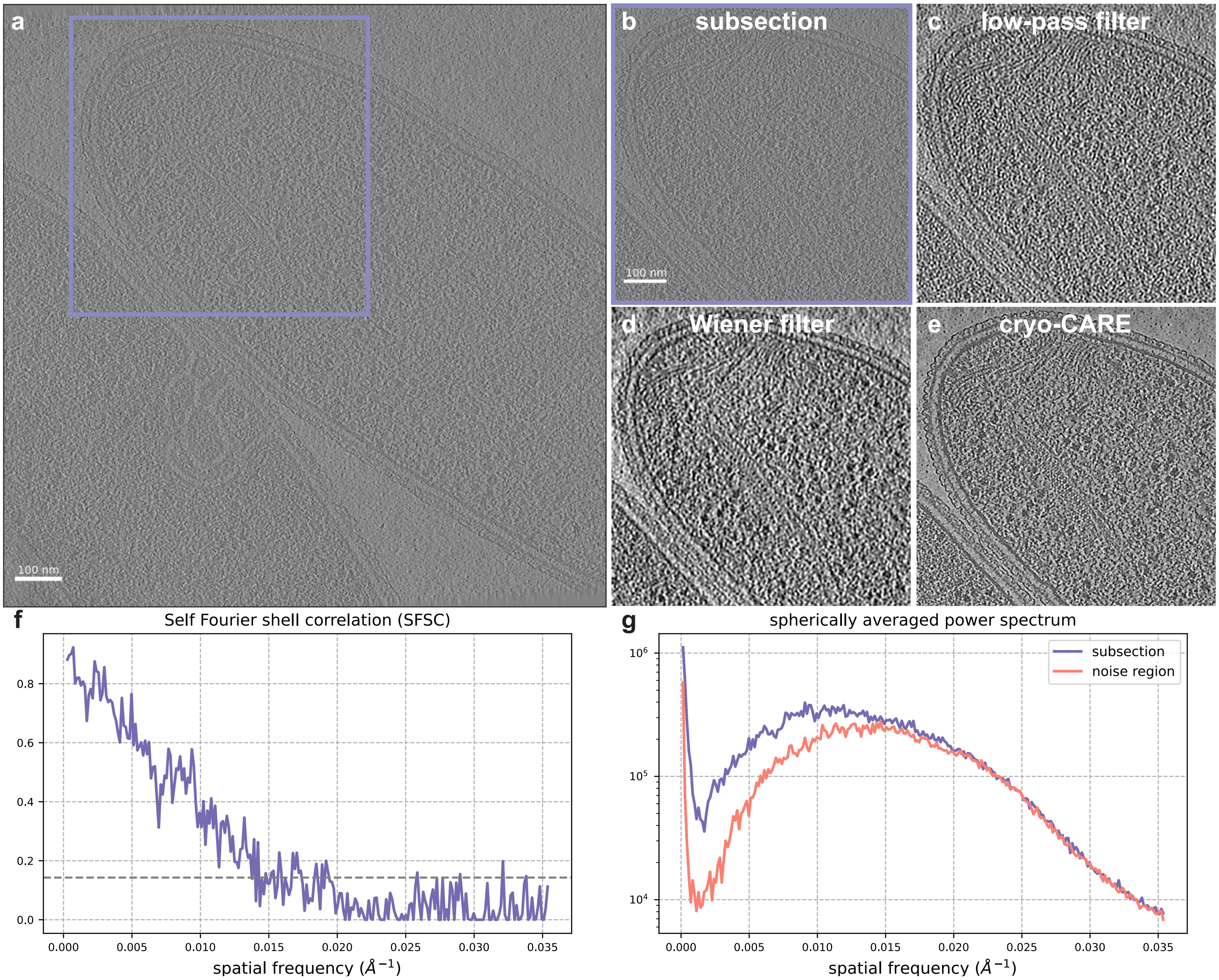

Figure S6: Comparison of denoising methods for a subsection of the tomogram EMD-15056 [31]. **(a)** Slice of the reconstructed tomogram ($N \times N = 928 \times 928$, pixel size $= 14.1$Å). **(b)** Region of interest from a subsection of the tomogram ($N \times N = 500 \times 500$). **(c)** Low-pass filter of the subsection at 72Å, corresponding to the 1/7 threshold in the SFSC. **(d)** Slice from the region of interest after applying a Wiener filter. **(e)** Slice from the region of interest denoised using cryo-CARE. Images in (c-e) are displayed at a threshold of ±2 standard deviations of the pixel values. **(f)** SFSC computed from the tomogram subsection. **(g)** Spherically averaged power spectrum of the region of interest slice and the background noise slice. While the Wiener filter improves contrast for features like membrane edges and ribosomes, cryo-CARE excels at both suppressing background and enhancing relevant high frequency information.
